# Overwhelming Post-Splenectomy Infection Syndrome: Variability in Timing With Similar Presentation

**DOI:** 10.7759/cureus.9914

**Published:** 2020-08-21

**Authors:** Anupam K Gupta, Oscar A Vazquez

**Affiliations:** 1 Minimally Invasive Surgery, University of Miami Hospital, Miami, USA; 2 Surgery, Charles E. Schmidt College of Medicine, Florida Atlantic University, Boca Raton, USA

**Keywords:** opsi, splenectomy, infection, sepsis

## Abstract

Overwhelming post-splenectomy infection (OPSI) syndrome is a rare and well-known entity that rapidly progresses with poor outcomes. Two patients underwent splenectomy after trauma and later presented with flu-like symptoms and thrombocytopenia, which then progressed to fulminant sepsis and death. The first patient had sepsis 20 days post-splenectomy, and the second patient underwent splenectomy 15 years before presentation. Both patients expired within 24 hours of the onset of symptoms. Even with no specific criteria for diagnosis, prompt identification of the overwhelming post-splenectomy infection is necessary; however, the prognosis is usually poor, even with aggressive treatment.

## Introduction

Splenectomy is performed as a life-saving surgery in the trauma setting as the spleen is the most commonly injured organ in blunt abdominal trauma (up to 31%-50% of cases) [1,2]. There has been a decrease in the number of splenectomy patients using angioembolization, but a subset of patients who fail angioembolization still need a splenectomy if bleeding is present from secondary splenic rupture [3,4]. Overwhelming post-splenectomy infection (OPSI) syndrome is a well-known entity that is rare and fatal due to its rapidly progressive and fulminant course over 12-24 hours after the first sign of "flu-like" symptoms [5,6]. Although the first instances of bacterial sepsis occurring post-splenectomy were first documented in 1952 by King and Shumacker in infants and children, it is known to affect asplenic adults [7,8].

## Case presentation

Case #1

**Figure 1:**
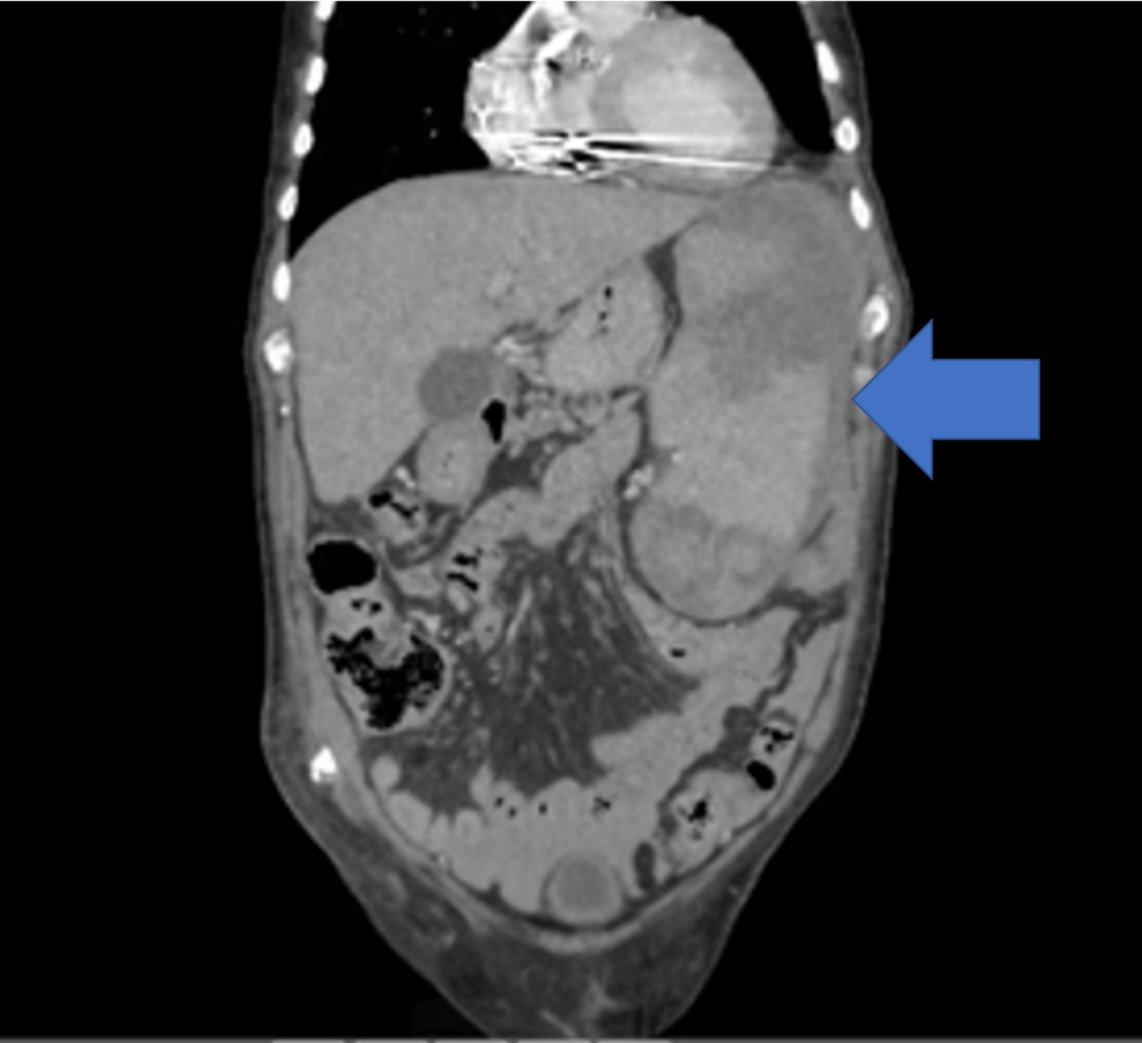
Computed tomography scan showing splenomegaly and splenic laceration

An 80-year-old male patient with a past medical history of a myeloproliferative disorder, coronary artery disease, and diabetes presented to the emergency room after a motor vehicle collision. On arrival, the patient's vital signs were significant for tachycardia of 115 beats per minute and blood pressure of 100 mm Hg systolic. The primary survey was unremarkable, and a secondary survey showed bruises and abrasions on the left side of the abdomen. A bedside focused ultrasonography for assessment of trauma (FAST) exam was positive for free fluid in the abdomen. The patient's vital signs stabilized with a one-liter bolus of Ringer's lactate solution. The patient then underwent a CT scan of the abdomen and pelvis, which showed splenomegaly and a splenic laceration (Figure 1). The patient underwent immediate angioembolization for splenic bleed. The patient was monitored for trends in hemoglobin, which showed a gradual decline with increasing abdominal pain, and this prompted the team to perform a splenectomy with a washout of blood clots. The postoperative course was uneventful, with the patient ambulating and tolerating a regular diet. Surgical staples were removed on day fourteen, and he was vaccinated for *Hemophilus influenza*, Streptococcal pneumonia, and *Neisseria meningitides.* The patient was scheduled to be transferred to a rehab facility when he complained of non-specific symptoms of body aches and nasal congestion. The clinical exam was remarkable only for a low-grade fever of 100.5 degrees Fahrenheit. Blood work significant values were thrombocytopenia of 20,000/mL (the normal value is 150,000-400,000/mL) and white blood cell count of 7,000/mL (the normal value being 4,500 to 11,000 WBCs/mL). The patient was initiated on broad-spectrum antibiotics because of fever, and cultures were drawn from blood, sputum, and urine. A chest x-ray and CT scan of the abdomen and pelvis revealed no foci of infection or fluid collection around the operative site. Over the next few hours, the patient showed increasing tachycardia and a drop in his mean arterial pressure to 60 mmHg from 80 mmHg. The patient was then monitored in the intensive care unit for vasopressor support with a sharp decline in clinical status. The patient became tachycardic to 120 beats per minute sinus rhythm, tachypneic at 20 breaths per minute, and hypotensive. The patient was intubated, supported with increasing vasopressors and was given steroids and platelets. The patient then progressed in acidosis and needed multiple cycles of cardiopulmonary resuscitation. The patient expired within 20 hours from the identification of thrombocytopenia. Blood cultures showed evidence of *Klebsiella pneumoniae* and *Escherichia coli* in both peripheral and central draws.

Case #2

**Figure 2:**
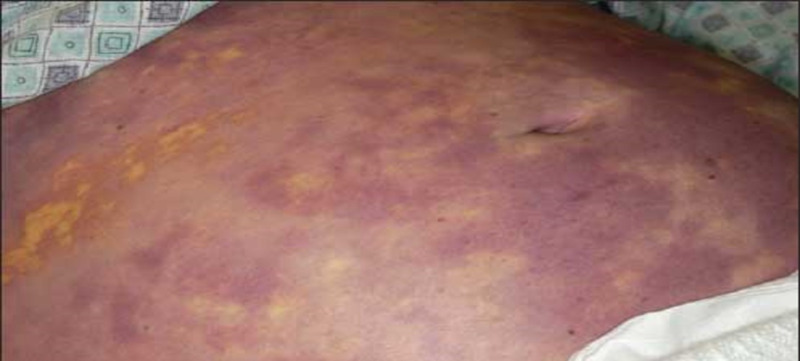
Rashes on patient’s abdomen

**Figure 3:**
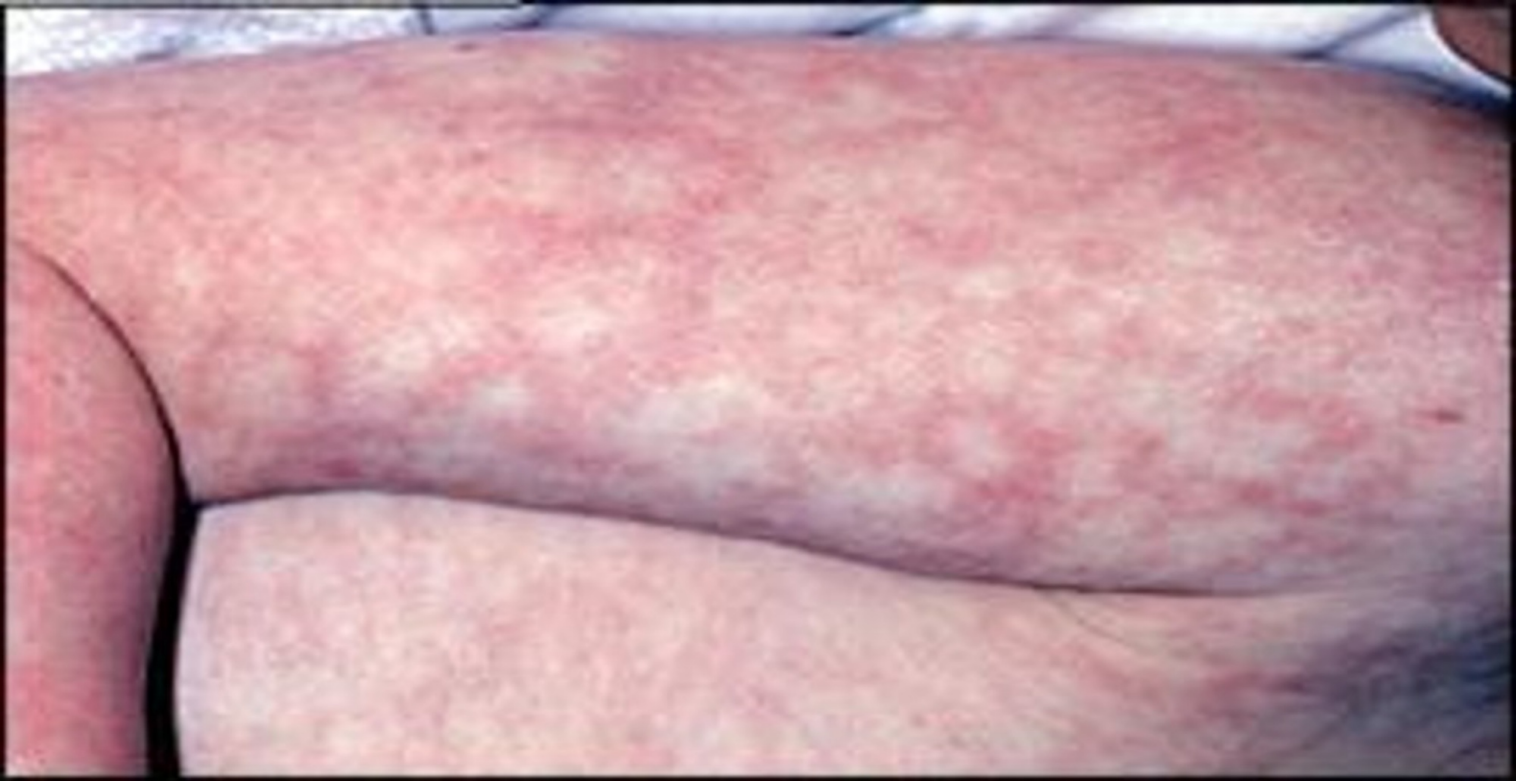
Rashes on patient’s right arm

A 54-year-old female patient with a past medical history of Hepatitis C infection, diabetes mellitus, hyperlipidemia, and hypertension presented with non-specific symptoms of mild weakness, lethargy, and development of rashes which began the day before. Her surgical history was remarkable for splenectomy 15 years prior due to trauma sustained in a motor vehicle collision. She was alert and conscious; however, vital signs showed tachycardia to 110 beats per minute, tachypnea of 35 breaths per minute, and systolic blood pressure of 100 mmHg, which responded after a fluid bolus to 115/74 mmHg. A detailed clinical exam was significant for rashes over her body (Figures 2, 3). Her blood work was remarkable for a white blood cell count of 33000/mL, thrombocytopenia of 20000/mL, INR 2.2 (normal being 1.1 or below), PTT 56 seconds (normal value are typically 25 to 35 seconds), fibrinogen 67 g/L (normal range is 2.0 to 4.0 g/L), creatine 2.3 mg/dL (normal range for creatinine in the blood is 0.84 to 1.21 mg/dL) and lactic acidosis of 11 mmol/L (normal blood lactate concentration in unstressed patients is 0.5-1 mmol/L). Patients' arterial blood gas analysis showed metabolic and respiratory acidosis with a pH of 7.29, bicarbonate of 17 mEq/L, PaCO2 of 70 mmHg, and a PaO2 of 80 mmHg. The patient was intubated and underwent a CT scan of the head, chest, abdomen, and pelvis, which revealed pneumatosis in the portal vein for which she underwent an emergent exploratory laparotomy. No pathological finding was seen on exploratory laparotomy, and the patient was immediately transferred with an open abdomen for further resuscitation in the intensive care unit. The patient was continued on blood products and close hemodynamic monitoring with a progressive increase in vasopressor requirements and acidosis over the next few hours. The patient expired over the next few hours despite multiple resuscitation efforts, and her blood, urine, and sputum cultures were unrevealing.

## Discussion

OPSI syndrome has a mortality rate of up to 50%, with a prevalence of 0.1%-0.5% [9]. Though the likelihood of this condition manifesting is most likely within three years from splenectomy, there have been reported cases up to 20 years from splenectomy, five years after the second patient presented had their spleen removed [7]. Common pathogens include Streptococcus pneumoniae, Haemophilus influenzae type b, group B Streptococcus, Staphylococcus aureus, Salmonella species, Escherichia coli, and other coliforms, Capnocytophaga canimorsus, and Pseudomonas aeruginosa [10]. OPSI syndrome classically begins with "flu-like symptoms" described as a prodrome composed of fever, chills, myalgia, headache, vomiting, and abdominal pain which then rapidly progresses to coma, acidosis, respiratory distress, septic shock, and disseminated intravascular coagulation (DIC) within 24-48 hours [11-13]. It is theorized that the cause of DIC and thrombocytopenia in patients infected with Streptococcus pnemoniae comes from polysaccharide-specific antibodies that activate the complement pathway and promote the deposition of complement fragments directly on to the bacterial capsule to cause thrombotic occlusion [14]. Other theories behind the pathogenesis of OPSI and its fulminant course include the loss of splenic phagocytic function, suppression of lymphocyte sensitivity, and the decreasing serum immunoglobulin levels causing a change in the opsonin system [15]. In later stages, the presentation is similar to that of Waterhouse-Friderichsen syndrome (WFS) with petechial rash and neurological manifestations with autopsy reports indicating the finding of bilateral adrenal hemorrhage [16,17].

Though OPSI syndrome is challenging to diagnose due to a lack of clear diagnostic criteria, clinicians should be extra vigilant in patients with a history of splenectomy presenting with fever, chills, diarrhea, and vomiting. Furthermore, in patients with signs of sepsis or septic shock, at least two sets of blood cultures should be drawn immediately before prompt administration of empiric, broad-spectrum antibiotics. Recommended laboratory investigations in these cases include a complete metabolic panel, liver function tests, blood glucose level, and serum lactate concentration. It is also recommended to draw a peripheral blood smear for the presence of bacteria and Howell-Jolly bodies as a result of asplenia [18]. Kumar et al. found that immediate administration of antibiotics increases survival rates and also recommend pneumococcal, Haemophilus influenza type b (Hib), meningococcal, and annual influenza vaccinations in asplenic individuals [19]. Treatment of OPSI is generally aggressive with intravenous fluids, antibiotics, vasopressors, steroids, heparin, packed red blood cells, platelets, cryoprecipitates, and fresh frozen plasma [15]. Due to its fulminant and fatal nature, prevention is of utmost importance in immunocompromised asplenic patients [5]. Recommended broad-spectrum antibiotics for asplenic patients include amoxicillin-clavulanic acid, trimethoprim-sulfamethoxazole (TMP/SMX), and cefuroxime since penicillins are now less-effective against increasing bacterial resistance [20]. Our patient's splenectomies were less than three weeks to fifteen years before presentation, yet they presented similarly with "flu-like" symptoms and thrombocytopenia. The first patient had a positive culture for encapsulated gram-negative organisms, and the second patient had a rash consistent with WFS. Together, they are an example of how rapidly this disease progresses and the variability in timing regarding the presentation post-splenectomy.

## Conclusions

OPSI syndrome may progress quickly, leading to fatal septic shock. The initial presentation can be non-specific flu-like symptoms with blood work significant for thrombocytopenia. It may also be caused by encapsulated gram-negative organisms like *Escherichia coli* and *Klebsiella* species, and, at times, there is no evidence of source on imaging and cultures. Finally, OPSI syndrome can occur in the immediate postoperative period to many years later.
